# Deficiencies in public understanding about tobacco harm reduction: results from a United States national survey

**DOI:** 10.1186/s12954-015-0055-0

**Published:** 2015-07-02

**Authors:** Marc T. Kiviniemi, Lynn T. Kozlowski

**Affiliations:** Department of Community Health and Health Behavior, University at Buffalo, State University of New York, School of Public Health and Health Professions, 3425 Main Street, Buffalo, NY 14214 USA

**Keywords:** Tobacco harm reduction, Cigarettes, Smokeless tobacco, Electronic cigarettes, Public health education, Risk perception, Health communication

## Abstract

**Background:**

Tobacco products differ in their relative health harms. The need for educating consumers about such harms is growing as different tobacco products enter the marketplace and as the FDA moves to regulate and educate the public about different products. However, little is known about the patterns of the public’s knowledge of relative harms.

**Methods:**

Data were analyzed from the Health Information National Trends Survey (HINTS) 4 Cycle 2, a population-representative survey of US adults conducted between October 2012 and January 2013 (*N* = 3630). Participants reported their perceptions of the relative risks of e-cigarettes, smokeless tobacco, and different types of cigarettes compared to “traditional” cigarettes. Relative risk perceptions for each product type, as well as the consistency and accuracy of harm reduction beliefs, were analyzed.

**Results:**

About 65 % of the respondents accurately reported that no cigarettes were less harmful than any others. Slightly more than half of US adults perceived e-cigarettes to be safer than regular cigarettes, a belief in line with current scientific evidence. By contrast, only 9 % of respondents perceived some smokeless tobacco products to be safer, a belief strongly supported by the evidence. Only 3.5 % of respondents had patterns of relative risk perceptions in line with current scientific evidence for all three modalities.

**Conclusions:**

The discrepancy between current evidence and public perceptions of relative risk of various tobacco/nicotine products was marked; for most tobacco types, a large proportion of the population held inaccurate harm reduction beliefs. Although there was substantial awareness that no cigarettes were safer than any other cigarettes, there could be benefits from increasing the percentage of the public that appreciates this fact, especially among current smokers. Given the potential benefits of tobacco risk reduction strategies, public health education efforts to increase understanding of basic harm reduction principles are needed to address these misperceptions.

## Introduction

Combusted tobacco products inhaled into the lungs are the greatest cause of health-related harms resulting from use of tobacco products [1, 2]. It is also acknowledged by the United States Food and Drug Administration (FDA) [3] and others [1, 4, 5] that there is evidence of a continuum of risk, with cigarettes constituting the most harmful tobacco/nicotine product and with nicotine replacement products, some smokeless tobacco products, and electronic cigarettes involving relatively low levels of risk [3, 6]. Though not welcomed by all tobacco control authorities (e.g., [7]), there has been support for including harm reduction strategies as one of the many traditional strategies for reducing the death and disability caused by tobacco use, particularly cigarette smoking (e.g., [2, 8–10]). Tobacco harm reduction focuses on encouraging the use of less dangerous forms of tobacco/nicotine by those who prefer not to abstain from all tobacco/nicotine products (e.g., [4]).

The FDA through its Center for Tobacco Products intends to invest in public education campaigns to “help educate the public--especially youth--about the dangers of regulated tobacco products” [11]. Coupled with a proliferation in the marketplace of “alternative” tobacco products (e-cigarettes, hookah, smokeless formulations) that differ in degree of risk [6] and the influence of perceptions of risk on product usage [12], it could be important for the FDA to help inform the public of the differential risks of the use of various tobacco/nicotine products [13].

Given the scientific consensus that cigarettes are the most deadly form of tobacco use, the public has a right to a clear understanding of this fact [14–16] and efforts should be made to impart an understanding of the differential health risks for various tobacco/nicotine products [13]. In order to accomplish this goal, though, we need to know how the public’s understanding matches current scientific knowledge about relative harms. Given that information about risk is used to make behavioral decisions [12], such knowledge about public perceptions of risks will help us better understand choices that individuals make about tobacco product use. Moreover, given the ethical obligation to communicate accurate information about harm reduction [14] and the FDA’s plans to educate the public about tobacco, understanding where the public is at now with respect to risk beliefs about tobacco products is an important step toward designing effective programming.

To advance these goals, we examined public perceptions of the relative risk of different tobacco formulations using data from the National Cancer Institute’s Health Information National Trend Survey (HINTS) 4 Cycle 2 [17]. The survey included questions about the risks, relative to “traditional” cigarettes, of different types of cigarettes, smokeless tobacco products, and electronic cigarettes. Tan and Bigman [18] have reported results for e-cigarettes from this survey but did not explore interrelationships across the multiple tobacco harm reduction questions. We used these questions to examine the current distribution of relative risk knowledge and, based on current epidemiological understanding (reviewed below), to determine how public perceptions match to the scientific evidence.

### Evidence for harm reduction modalities

The alternative tobacco product modalities addressed in the HINTS survey can be organized according to the clarity of what the correct, evidence-based answer should be regarding their riskiness relative to smoking “traditional” cigarettes. The question (see “Methods” for the questions) asking whether some cigarettes are less harmful than others is correctly answered, “no.” The argument for cigarettes being equal in risk is made here [19]. A key principle for understanding this issue is to understand the problem of compensatory smoking, in which smoking behavior can cause smokers to get what they desire from any brand of conventional cigarette based on how they puff on the cigarette (e.g., [20]).

Second, the question of whether some smokeless tobacco products are less harmful than cigarettes is correctly answered, “yes.” While not safe, some smokeless tobacco formulations are significantly less dangerous to users than cigarettes [21]. One of the most thorough reviews was done for the European Union [22] and concluded, referring to major chronic diseases that “. . . . in relation to the risks of the above major smoking-related diseases, and with the exception of pregnancy, [smokeless tobacco products] are clearly less hazardous, and in relation to respiratory and cardiovascular disease substantially less hazardous, than cigarette smoking.” (p. 114–115). For cardiovascular disease, the risk reduction was judged as “at least 50 %,” for oral and gastrointestinal cancer “probably also at least 50 %,” and for respiratory disease “close to 100 %.”

Finally, for electronic cigarettes (e-cigarettes, vaping), the question asked if they were more or less harmful than cigarettes; the best evidence-based opinion at the present time would be, not safe, but much lower in risk [23, 24].

### Public perceptions and patterns of responses on harm reduction

We examined descriptive statistics on the proportions of individuals holding harm reduction beliefs about each formulation, as well as the “match” of the public’s beliefs and the evidence-based answers reviewed above. In addition to assessing beliefs question by question, the coherence or consistency of accurate beliefs across questions is important, because it could indicate a broader understanding of the underlying etiological principles involved.

## Methods

The Health Information National Trends Survey 4 Cycle 2 dataset was analyzed. HINTS is a nationally representative survey of US adults. The Survey 4 Cycle 2 data collection took place between October 2012 and January 2013. A mailed paper and pencil questionnaire (available in both English and Spanish) was used to collect data. The study design and sampling framework have both been described in detail elsewhere [17]. Briefly, HINTS uses a two-stage sampling design. The first stage consists of a stratified sampling procedure based on US residential addresses to select households for inclusion. The second stage uses the next birthday method to select an individual adult in each household to complete the study. The overall response rate was 39.9 %, resulting in an *N* = 3630 for completed questionnaires.

### Measures

#### Harm reduction beliefs

Beliefs about the relative harms of different types of cigarettes were assessed with the question: “In your opinion, do you think that some types of cigarettes are less harmful to a person’s health than other types?” Answer options were Yes, No, Don’t Know. For smokeless tobacco, relative harms were assessed by asking “In your opinion, do you think that some smokeless tobacco products, such as chewing tobacco, snus and snuff are less harmful to a person’s health than cigarettes?” with answer options of Yes, No, Don’t know. Finally, for electronic cigarettes (e-cigarettes, vaping), the question asked “New types of cigarettes are now available called electronic cigarettes (also known as e-cigarettes or personal vaporizers). These products deliver nicotine through a vapor. Compared to smoking cigarettes, would you say that electronic cigarettes are_______?” and included a Likert-type response scale (1 = much less risk to 5 = much more risk).

#### Smoking behavior

Two questions assessed smoking behavior. First, ever smoking behavior was assessed with the question “Have you smoked at least 100 cigarettes in your entire life?” Those answering yes were then asked about current smoking behavior with the question “How often do you now smoke cigarettes?” with response options of 1 = everyday, 2 = some days, 3 = not at all. The combination of these two questions was used to characterize respondents as never, former, or current smokers, with individuals who reported never smoking 100 cigarettes classified as “never” smokers, individuals who reported having smoked at least 100 lifetime cigarettes but currently smoking not at all classified as “former” smokers, and individuals who reported current smoking every day or some days classified as “current” smokers.

### Analysis strategy

Analyses were conducted using Stata version 14 (Stata Corp., College Station, TX). All analyses were done using Stata’s complex survey design commands and used the HINTS final survey and jackknifed replication weights. These weights account for sampling design, oversampling, and non-response patterns. Descriptive statistics were used to characterize the population distributions of responses to each relative risk question and of the overall number of harm reduction beliefs. For examining the relation of smoking status to harm reduction beliefs, we utilized Rao and Scott’s method for calculating F-test adjusted statistics for complex survey data [25, 26]. When omnibus tests were significant, we then conducted follow-up paired comparisons comparing different combinations of smoking status using the same statistical procedures. Finally, to test for coherence/consistency of harm reduction beliefs across types of tobacco products, we coded each harm reduction belief as “accurate” or “inaccurate”—accurate was defined as (a) no cigarettes are safer, (b) smokeless is safer, and (c) e-cigarettes are safer. We then used descriptive statistics to characterize the proportion of the population holding each combination of beliefs.

## Results

As described above, HINTS is a US nationally representative survey and all analyses were done using survey weighted analysis techniques. Therefore, the demographic characteristics relevant to the results reported here are those of the US adult population.

### Are some cigarettes safer? No

Table 1 presents descriptive data for perceptions of the relative risk of different types of cigarettes. Only 13 % of respondents believed that some types of cigarettes were less harmful than other types. This perception differed by smoking status, *F*(3.61, 176.86) = 5.68, *p* < .01. Current smokers and former smokers were both more likely than never smokers to perceive lower risks; never versus former *F*(1.98, 96.89) = 3.54, *p* < .05; never versus current *F*(1.98, 96.81) = 7.59, *p* < .001. Current smokers were also more likely than former smokers to perceive lower risk; former versus current *F*(2.00, 97.95) = 3.06, *p* < .05.

**Table 1:** Harm reduction beliefs for different types of cigarettes and for smokeless tobacco products (SLT), *N* = 3630

	Overall (percent of population (95 % CL))		By smoking status (percent of population (95 % CL))					
			Never smokers		Former smokers		Current smokers	
	Cigs	SLT	Cigs	SLT	Cigs	SLT	Cigs	SLT
Yes	12.6 %	9.4 %	9.9 %	9.2 %	12.5 %	9.7 %	21.9 %	10.0 %
	(10.6, 14.9)	(7.7, 11.5)	(8.0, 12.2)	(7.1, 11.8)	(8.7, 17.5)	(7.7, 12.2)	(15.4, 28.9)	(5.8, 16.6)
No	64.9 %	73.5 %	64.1 %	70.1 %	69.0 %	77.3 %	62.0 %	76.8 %
	(62.0, 67.6)	(70.9, 75.9)	(60.5, 67.5)	(66.7, 74.6)	(63.6, 73.9)	(73.6, 80.1)	(52.7, 70.5)	(69.0, 83.1)
Don’t Know	22.6 %	17.1 %	26.0 %	19.9 %	18.6 %	13.0 %	16.6 %	13.2 %
	(20.5, 24.8)	(15.1, 19.4)	(22.7, 29.7)	(16.9, 23.2)	(14.9, 22.9)	(10.4, 16.1)	(11.2, 24.0)	(8.4, 20.2)

### Are any smokeless products safer than cigarettes? Yes

Table 1 presents beliefs about the relative risks of smokeless tobacco relative to cigarettes. Less than 10 % of respondents perceived smokeless tobacco as less risky than cigarettes. There were not differences in risk perception by smoking status; *F*(3.27, 160.42) = 2.28, *p* = .07. In other words, about 9 in 10 individuals did not know that smokeless tobacco products are less hazardous than cigarettes.

### Are e-cigarettes safer than cigarettes? Yes

Descriptive statistics for the perceived relative risk of e-cigarettes versus regular cigarettes are presented in Table 2. Proportions in this table are based on the 77 % of respondents who had heard of e-cigarettes. As can be seen in the table, just over half of the general population (51 %) believe e-cigarettes to be less harmful than regular cigarettes. This relative risk perception differs by smoking status, *F*(6.32, 309.55) = 4.41, *p* < 0.001. Current smokers were more likely to perceive e-cigarettes as less dangerous than were either former or never smokers; never versus current *F*(3.22, 157.70) = 8.05, *p* < .001; former versus current *F*(3.69, 181.00) = 4.31, *p* < .01. Never smokers and former smokers did not significantly differ, *F*(3.51, 171.76) = 1.04, *ns*.

**Table 2:** Beliefs about the relative risks of e-cigarettes versus “regular” cigarettes, overall and by smoking status, *N* = 3630

Perceived relative risk	Overall (percent of population (95 % CL))	By smoking status (percent of population (95 % CL))		
		Never smokers	Former smokers	Current smokers
Ecigs Much less harmful	11 %	8.5 %	12.2 %	16.3 %
	(9.2, 13)	(6.5, 11)	(8.9, 16.7)	(12.1, 21.4)
Ecigs Less harmful	40 %	37.3 %	37.2 %	48.7 %
	(36, 43)	(33.5, 41.4)	(32.1, 42.6)	(40.8, 56.8)
Ecigs Just as harmful	46 %	50.7 %	48.2 %	33.2 %
	(43, 49)	(46.6, 54.7)	(42.7, 53.7)	(26.1, 41.1)
Ecigs More harmful	1.6 %	1.9 %	1.4 %	1.2 %
	(1.0, 2.6)	(0.9, 3.7)	(0.7, 2.7)	(0.5, 2.8)
Ecigs Much more harmful	1.2 %	1.6 %	0.9 %	0.6 %
	(0.7, 2.3)	(0.7, 3.5)	(0.3, 3.6)	(0.2, 1.6)

### Coherence of accurate harm reduction beliefs

Finally, with respect to patterns and coherence of harm reduction beliefs, patterns of responses for participants who answered all three harm reduction questions were examined. This analysis includes approximately 70 % of the sample. Because the e-cigarette risk question was preceded by a screener asking participants if they had ever heard of e-cigarettes and the risk question was only asked of those who said yes, the sample for this analysis drops to 70 %, given that this analysis requires valid responses on all three of the relative risk questions.

Only 3.5 % agree with the most coherent accurate view of tobacco harm reduction that (a) no cigarettes are safer, (b) smokeless is safer, and (c) e-cigarettes are safer. Beyond that, 1.9 % agree only no cigarettes and smokeless are safer, but disagree on e-cigarettes being safer; 38 % agree only no cigarettes and e-cigarettes are safer, but disagree on smokeless being safer; 44 % only think no cigarettes are safer; 0.8 % only think smokeless is safer; 7.7 % only think e-cigarettes are safer; and 3.1 % who completely fail at having any accurate harm reduction belief.

## Discussion

Clearly, the public does not show an expert understanding of tobacco/nicotine harm reduction. These limitations in the public’s understanding have the potential to lead to both individual and public health harms. For example, the finding here that 75 % of US adults hold the misperception that smokeless tobacco products are just as harmful as cigarettes is both a considerable lack of knowledge and a serious public health problem. If a smokeless tobacco user, for example, believes that smokeless tobacco products carry higher oral cancer risks than cigarettes, and therefore switches to cigarettes, this is objectively a step toward greater harm rather than reduced harm. One of the official warnings from the FDA on smokeless tobacco is “WARNING: This product is not a safe alternative to cigarettes.” This message may contribute to the perception of harm from smokeless tobacco. While “not safe,” it is clearly proven that smokeless tobacco products can be much safer than cigarettes [15]. However, our findings, in conjunction with earlier US surveys found similar ignorance about harm reduction from smokeless tobacco [27].

With respect to different types of cigarettes, although the majority of US adults believe that the risks are equal, there is room for concern for the 35 % overall who did not know that various cigarettes are equal in risk. And it is concerning that current smokers (21.9 %) were about twice as likely as former smokers (12.5 %) to think some cigarettes are safer than others. Although the FDA has banned the “light” and “mild” descriptors on cigarettes, there appears to have been no change in the use of filter ventilation on lower-tar cigarettes which help make them taste lighter and milder and contribute to the perception of reduced risk [28].

These findings strongly suggest the need for accurate and effective public health messaging around the relative harms of different tobacco products. There is reason to believe that such messaging could be successful. With respect to types of cigarettes, counter-marketing of lower-tar cigarettes has been successful in the past and should be considered again [29, 30]. Similarly, we know that harm reduction beliefs about smokeless tobacco (snus) do influence the use of these products [31, 32]. With respect to the etiology of tobacco-caused health problems and the role of different delivery systems, some basic principles (e.g., avoiding combustion-smoke in the lungs) would likely be easy to inform the public about [33]. Others have noted the importance for public education as part of the implementation of harm reduction policies [34, 35]. In addition to public health messaging, accurate physician knowledge of the relative risks of tobacco products could also be an important source of advice for smokers [31]. This is especially important because media reports have not helped clarify the risks of smokeless tobacco compared to cigarettes [36].

Results from another national survey found between 40 and 46 % of adults reporting that e-cigarettes are less harmful than cigarettes, with whites, current smokers, young adults, and those with at least a high-school diploma being the most likely to perceive reduced harm [37]. Asking a direct question that compares the risk of snus and cigarettes produces lower estimates of the prevalence of reduced harm perceptions than does the comparison of answers to separate questions on the risk of snus and on the risk of cigarettes [38], although the comparative question may be more consistent with the cognitive processing of comparative risk [39].

There are some barriers to accurate and effective messaging on this topic. Currently the FDA regulations present significant barriers for manufacturers to make cross-product risk comparisons [40]. The value of these barriers need to be weighed against the possible ill effects of beliefs that are currently held by the public and that can influence behavior. Note the FDA regulations have concerns about promoting reduced-risk products that might increase the population level of use of this safer product. Consumers themselves, however, can be interested in their own levels of toxicological risk—no matter the effects on population health. When some products, like e-cigarettes and snus, are so much less dangerous than cigarettes, it becomes unlikely that increased levels of use could ever produce a net population health loss in comparison to cigarettes [41].

One effective model might be the United States National Institute on Alcohol Abuse and Alcoholism (NIAAA). NIAAA provides information on alcohol use that can help better understand the risks of drinking and the differences between beverages [42]. The FDA’s plans to provide public education on tobacco/nicotine products should include informing the public of some rudiments of how tobacco harm reduction occurs (e.g., avoiding inhalation of combusted products) and develop ways to go beyond the serious limitations of informing the public that “no product is safe” [15].

There are, of course, limitations to the work that should be considered. First, the survey of necessity captures beliefs of the public at one particular time point. Especially in the context of newer tobacco product formulations, public awareness, knowledge, and risk perceptions are likely to be dynamic. Ongoing assessment of these perceptions and examination of changes over time would be a valuable addition to the literature. Second, risk perceptions may be at least somewhat dependent on the wording of particular questions. For example, the “types of cigarettes” question is ambiguous as to what qualifies as different types of cigarettes. There is, therefore, the possibility that variability in how individuals interpret the question might affect their risk perceptions.

## Conclusions

The discrepancy between current evidence and public perceptions of relative risk of various tobacco/nicotine products was marked. Even for e-cigarettes, half of the public incorrectly believed them to be just as dangerous as cigarettes and an overwhelming majority of respondents incorrectly believed smokeless tobacco to be just as dangerous as traditional cigarettes. Although there was substantial awareness that no cigarettes were safer than any other cigarettes, there could be benefits from increasing the percentage of the public that appreciates this fact, especially among current smokers. Given the potential benefits of tobacco risk reduction strategies, public health education efforts to increase understanding of basic harm reduction principles are needed to address these misperceptions.
